# The Role of Live and Dead Corals in Shaping Fish Assemblages Across Life Stages

**DOI:** 10.1002/ece3.72443

**Published:** 2025-11-12

**Authors:** Felipe M. G. Mattos, Aziz J. Mulla, Vianney Denis, Che‐Hung Lin, Tzu‐Hao Lin, Yoko Nozawa

**Affiliations:** ^1^ Biodiversity Research Center Academia Sinica Taipei Taiwan; ^2^ Department of Life Science National Taiwan Normal University Taipei Taiwan; ^3^ Biodiversity Program, Taiwan International Graduate Program Academia Sinica and National Taiwan Normal University Taipei Taiwan; ^4^ Institute of Oceanography National Taiwan University Taipei Taiwan; ^5^ Université Côte D'azur, CNRS UMR 7035 ECOSEAS Nice France; ^6^ Sesoko Station, Tropical Biosphere Research Center University of Ryukyus Okinawa Japan

**Keywords:** fish recruits, functionality, habitat loss, recovery, trophic ecology

## Abstract

Coral reef fishes face unprecedented threats, as extensive habitat degradation compromises their ecological functions by modifying assemblage structure. It remains unknown how resistant reef fishes are to widespread losses in coral cover, and most studies tend to focus on adults, overlooking the important role of recruits. This study employed taxonomic and trait‐based approaches to investigate how live and dead branching corals influence reef fish assemblages across life stages. Over 1 year, we monitored recruitment and the migration of post‐recruits (juveniles and adults) on manually constructed 1 m^2^ patches of live and dead branching corals in a degraded reef. Recruit assemblages, composed mainly of two trophic groups, exhibited similar abundance and richness in the complex structures of dead and live coral patches, compared to flat control patches. Conversely, post‐recruit fishes were more abundant, species‐rich, and functionally diverse in live coral patches, encompassing several trophic groups and displaying a dominance shift between mobile and sedentary species. Our findings reveal that while dead coral structures can serve as temporary shelters for mobile recruits, live corals are essential for supporting long‐term biodiversity and diverse functional traits. This study underscores the complementary roles of both live and dead corals in promoting reef fish recovery and highlights the value of integrative strategies for reef ecosystem restoration.

## Graphical abstract

We assessed the effects of experimental patches of live and dead corals on reef fish assemblages in different live stages. While dead and live corals provided equal shelter for mobile recruits, only live corals supported higher post‐recruit abundance, species richness, and functional diversity. These findings highlight the complementary roles of live and dead corals in fish recovery and stress the importance of integrative reef restoration strategies.

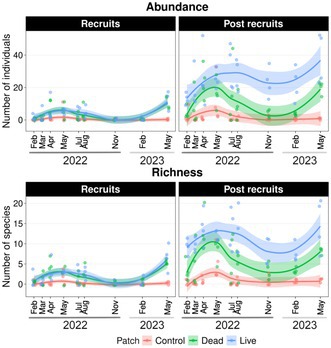

## 1 Introduction

In recent decades, fish assemblages along coral reefs have suffered extensively from habitat degradation triggered by anthropogenic activities (Bellwood et al. 2019; Garpe et al. 2006). This has led to a global decline in reef fish biodiversity (Munday 2004; Strona et al. 2021), biomass (Christensen et al. 2014), and the associated functions (Ainsworth and Mumby 2015; Bonin et al. 2011; Emslie et al. 2008; Jones et al. 2004; Morais and Bellwood 2020). Conversely, fishes are critical for reef dynamics, and changes in their assemblage structure may further precipitate coral reef decline (Brandl et al. 2020; Morais et al. 2022; Morais and Bellwood 2020; Mouillot et al. 2014), risking a negative loop.

Several key ecosystem processes in coral reef functioning involve fishes (Brandl et al. 2019; Mouillot et al. 2013). Herbivorous and planktivorous fishes play an important role in the transfer of energy from low to high trophic levels, boosting secondary productivity (Bellwood et al. 2018; Morais and Bellwood 2020; Tebbett et al. 2024). Furthermore, sedentary fishes exert a strong local influence by helping with nutrient recycling (Collins et al. 2024; Siqueira et al. 2021), while mobile species impact sediment distribution and nutrient transfer between habitats (Brandl et al. 2019; Tebbett et al. 2025). Additionally, healthy fish assemblages consist of diverse herbivorous fish populations that limit algal growth (Hughes 1994; Jessen and Wild 2013; Lewis and Wainwright 1985) and help maintain stable coral cover (Mumby 2006).

While several studies measured the effect of live colonies and the percentage of live coral cover on reef fishes (Bell and Galzin 1984; Chabanet et al. 1997; Sano et al. 1984), only a few studies have investigated the capacity of coral reef fish to use dead corals, mostly restricted to a single species or family (see Fakan et al. 2025; Streit et al. 2021; Tolimieri 1995; Wilson et al. 2006), with even fewer involving early life stages (e.g., Feary et al. 2007; Ohman 1990; Tolimieri 1995; Wismer et al. 2019). Due to the practical difficulties involved in studying small fishes in the field, many ecological surveys overlook recruits (e.g., Helder et al. 2022; Liu et al. 2025), thus failing to capture their ecological significance as the foundation for future assemblages (Halpern et al. 2005; Jones 1990; Sponaugle 2015). In many species, recruits inhabit distinct habitats (Félix‐Hackradt et al. 2014; Grol et al. 2014; Kimirei et al. 2013) and require different diets (Bellwood 1988; Chan et al. 2019) from adults, gradually adopting mature traits as they grow. The habitat use of recruits varies by taxa and environmental conditions, with some species depending on live corals (obligate live coral dwellers) (Bonin et al. 2009; Feary et al. 2007; Lecchini et al. 2013), while others prefer dead colonies or show no preference (facultative live coral dwellers) (Feary et al. 2007; Lirman 1994). Furthermore, ontogenetic development influences the degree of preference for specific habitats (Komyakova et al. 2019; Lecchini and Galzin 2005; Lirman 1994).

As degraded reef habitats become increasingly prevalent worldwide (see Bruno et al. 2019; Bruno and Selig 2007; De'ath et al. 2012), the capacity of dead corals to support a functionally diverse fish assemblage, compared to live corals, remains unclear. Furthermore, disturbances in coral reefs often lead to habitat fragmentation (Bonin et al. 2011), which accentuates the patchiness in these ecosystems (Bonin et al. 2011; McClanahan 2022), creating a mosaic of variable biomass and productivity rates within the same reef (Agudo‐Adriani et al. 2019; Syms and Jones 2000). Responses to coral mortality and habitat fragmentation depend on a combination of species traits, spatial distribution in reef fish assemblages, and recruitment pulses (Syms and Jones 2000). While some young, site‐attached damselfishes appear relatively resilient to disturbances and coral mortality (see Wismer et al. 2019), the role of dead corals in maintaining functional fish assemblages in patchy reef habitats remains overlooked.

To address these knowledge gaps, we evaluate how live and dead corals impact reef fish assemblages on successive life stages and how habitat variations influence functional traits. We hypothesize that recruit and post‐recruit (juveniles and adults) assemblages will exhibit different richness, abundance, and foraging traits in live and dead coral patches. To test this, we conducted a year‐long field experiment using patches of live and dead branching corals, comparing their associated reef fish assemblages to those observed along flatter degraded reef substrata.

## 2 Methods

### 2.1 Study Site

**FIGURE 1:**
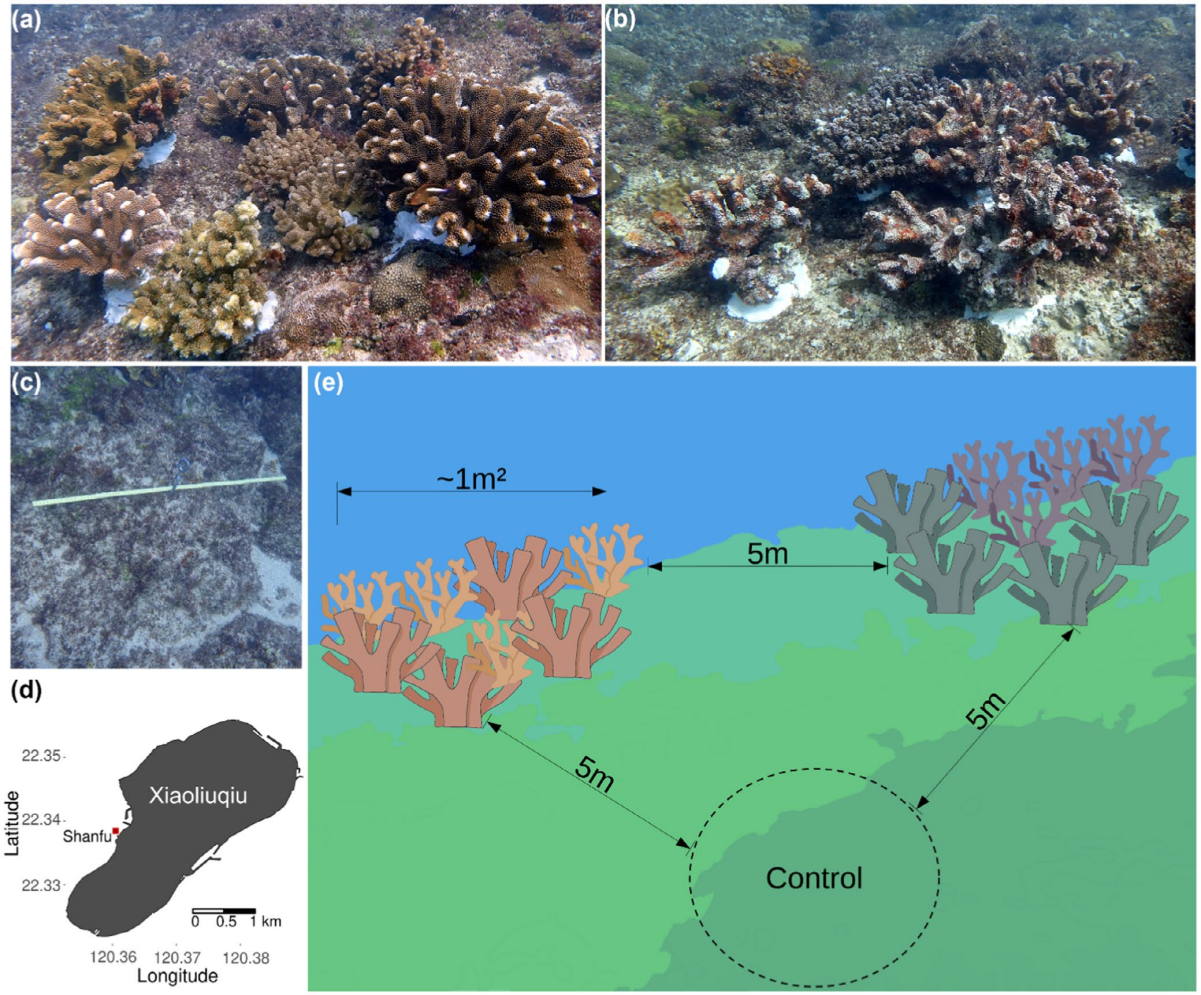
The present study employed four replicate blocks, each including three patches: a live coral treatment (a), a dead coral treatment (b), and a control (c), located near Shanfu harbor in Xiaoliuqiu (d). The 1 m^2^ patches were placed approximately 5 m from each other (e). The replicate blocks were placed approximately 10 m apart from each other.

This study was conducted at Xiaoliuqiu Island in southern Taiwan, 22°20′24″N120°22′12″E. This 6.8 km^2^ island was once home to diverse reefs with high coral and fish richness (Yang et al. 2017). However, intensive anthropogenic activities such as overfishing and coastal development have dramatically reduced biodiversity (Dai et al. 2009), causing severe declines in live coral cover (Lin et al. 2024; Yang et al. 2017). Despite a Marine Protected Area (MPA) established in 2000, with further zoning in 2014 prohibiting fishing activities, coral cover remains in decline, with no sign of recovery for both corals and the associated reef fish assemblage. To the west of the Island (Shanfu Harbor, Figure 1d), shallow‐water reefs extend approximately 500 m along the island's coastline and still host relatively high coral cover compared to other areas around the island (Lin et al. 2024; Mattos et al. 2025). This site has a shallow slope (1–10 m) dominated by turf algae (less than 5% coral cover) on a relatively flat, hard substrate (“turf zone”), while a deeper area (10–17 m) still maintains moderate coral cover (~30%) and a complex structure (“coral zone”). This allowed for a manipulation experiment to test the effect of patches of live and dead corals on shallow reef fish assemblages.

### 2.2 Data Collection

#### 2.2.1 Experiment Setting and Monitoring

Between February 2022 and May 2023, we conducted a field experiment in the turf zone using live and structurally intact dead coral colonies from two different branching species: *Pocillopora eydouxi* and *Pocillopora verrucosa* . All colonies were collected from the surrounding reef and transplanted to the experimental area. Colonies of *P. eydouxi* (~40–60 cm tall and ~40–70 cm wide) and *P. verrucosa* (~30–40 cm tall and ~40–60 cm wide) were carefully removed from their bases using a crowbar. To ensure that live and dead coral patches held comparable structural complexities, we brushed the macroalgae off the dead corals with steel wire brushes, exposing branches and simulating recently dead corals. The original resident fish and invertebrates were also carefully moved to a new substrate before transplantation. Each coral was then secured to the substrate with a 10 cm bolt and underwater epoxy. Four experimental blocks (i.e., four replicates) were constructed in the turf zone, each containing two experimental treatments (a live coral patch and a dead coral patch) and a flat reef area (control patch) (Figure 1a–c).

Treatment patches consisted of four colonies from each coral species, resulting in eight colonies per patch in a total area of approximately 1 m^2^. The patches were spaced ~5 m apart, forming a triangular shape in each replicate block (Figure 1e). The control patch was defined as a 1 m^2^ circle on the flat substrate, and a peg was used to mark its center. Experimental blocks were positioned approximately 10 m apart, parallel to the coastline, at a depth of ~5 m. After set‐up, the live and dead coral patches held a cluster of *Pocillopora* spp. colonies up to 5 cm apart and displayed higher vertical rugosity and more holes than the surrounding flat substrate (see Figure 1a–c). Control patches were dominated by the turf matrix but also displayed sparse holes and occasional small colonies. To compare patch habitat conditions over time, we measured live coral cover and height range, counted the number of refuge categories, and visually estimated rugosity, assigning ranks from 1 to 5, with 1 indicating the lowest and 5 the highest values. This ranking system captured temporal changes in patch structure, such as colony breakage and algal overgrowth. Detailed definitions of the rank scores are provided in Table A1.

The patches (treatment and control) were monitored monthly for fish composition for the first 6 months starting from February 2022, expecting recruitment pulses and a stabilization of assemblage dynamics within that time. Monitoring in June 2022 was canceled due to adverse weather conditions. From August 2022 onward, patches were monitored quarterly until May 2023 using an Underwater Visual Census (UVC) method adapted from Bohnsack and Bannerot (1986). In this approach, a diver swam around and above each patch for a maximum of 2 min, identifying and counting fish species foraging or sheltering in the patches. A trained researcher with over 10 years of experience in UVC and size estimation recorded the size of the recruits using a ruler for reference. For logistical reasons, a local collaborator conducted the field monitoring in April and July 2022 by recording 2 min Diver‐Operated Videos (DOV) with a ruler as a scale bar to emulate the UVC method. This method has been extensively used and produces abundance counts similar to those of the UVC method (Wilson et al. 2018). To test for differences between methods, we used Dunn's tests with Holm's correction for repeated measures.

Recruits, defined as recently settled individuals that have already transitioned to a demersal lifestyle (Jenkins et al. 2009; Keough and Downes 1982), were identified based on behavior, coloration, and the species‐specific settlement size. Individuals were classified as recruits if their length was up to 2 cm larger than the species' settlement size. This relative threshold was used to exclude older juveniles that had grown substantially post‐settlement. All larger juveniles—i.e., over 2 cm above the species' settlement size—and adults were grouped into one category called “post‐recruits,” as our goal was to focus on the different responses between naive recruits and more experienced fish. Such differences are often linked to ontogenetic changes in diet and mobility (Bellwood 1988; Chan et al. 2019; Chen 2002; Félix‐Hackradt 2013; Giffin et al. 2019), as well as size thresholds in mortality rates (Frederick 1997; Goatley and Bellwood 2016; Kimirei et al. 2013; Lewis 1997; Mccormick and Makey 1997). Thus, we opted to use these ecological differences linked to habitat use to group fish assemblages into either recruits or post‐recruits. Settlement size data were retrieved from the literature (Brothers et al. 1983; Leis 1984; Bellwood and Choat 1989; Wellington and Victor 1989; Thorrold 1993; Chen 2002; Sadovy et al. 2003; Juncker et al. 2006; Leis et al. 2011; Leu et al. 2012, 2022; Baensch 2016, 2025; Grutter et al. 2017) to the nearest taxonomic level (See Table A2). This approach was considered conservative due to the rapid growth rates of recruits (Booth and Hixon 1999; Leahy et al. 2015; Lou 1993). To compare temporal dynamics observed in the experimental patches and control zones to seasonal trends in the local ichthyofauna, we conducted quarterly surveys of fish assemblages in both the turf and the coral zones throughout the study. In each zone, three transect lines were randomly deployed 5–10 m apart, and fish were surveyed using a belt‐transect UVC method (30 m × 2 m) to count and identify the recruits and post‐recruits. All field procedures were approved by the Pingtung County Government (Permit No. 11130157900, issued on January 24, 2022).

#### 2.2.2 Species Traits

For our functional analyses, we selected two key traits: mobility and trophic group. These traits reflect the species' feeding habits and their foraging grounds. For example, ambush carnivores tend to be sedentary, while roving herbivores constantly swim around to forage (Agudo‐Adriani et al. 2019; Ferreira et al. 2015; Suzuki et al. 2018). Mobility mostly determines the species level of association with the substrate and their ability to move between habitats such as dead and live coral patches, therefore influencing habitat selection and use. Sedentary species usually have strong site fidelity and small home ranges (< 5 m), living in close association with the substrate, often with benthic organisms such as macroalgae, seagrass, sponges, or live corals (Waldner and Robertson 1980; Wilson et al. 2008). Mobile species have a wider home range (up to 100 m), exploring a greater number of habitats without being restricted to a particular substrate type (Chapman and Kramer 2000; Ferreira et al. 2015; Francini‐Filho et al. 2010; Tebbett et al. 2025). Mobility categories were based on the definitions from Donati et al. (2019), which simply categorized species into high and low mobility. Low mobility includes sedentary and territorial species, which may exhibit significant vertical movement but have limited horizontal mobility. High mobility includes species with home ranges spanning tens of meters.

Trophic group traits were based on the definitions of Ferreira et al. (2004), with the addition of “cleaners” (for species whose diets are primarily from cleaning other organisms) and “corallivores” (for obligate coral feeders). Cleaners occupy a specialized niche, interacting with different fish species, often choosing coral heads as cleaning stations to maximize their access to clients (Grutter and Poulin 1998). Corallivores differ from other sessile invertebrate feeders as they are generally associated with live corals, avoiding dead corals covered by algae (Brooker et al. 2016; Graham et al. 2009; Pratchett et al. 2006). In contrast, other sessile invertebrate feeders are often less specialized and feed on items available in a wider range of substrate types (Ferreira et al. 2004). Trophic trait data were obtained from FishBase (Froese and Pauly 2025; last accessed September 2025) and other published fish guides (Allen et al. 2015; Lieske and Myers 2001).

Trophic and mobility data were curated, and in cases where source information differed, we cross‐referenced publications on the feeding biology of the species to ensure the accuracy of the data. Trophic and mobility traits were assigned at the species level and were not life‐stage specific, as our goal was to examine potential trait‐based selection when comparing the effects of live and dead colonies on recruit and post‐recruit assemblages. This approach is correlative to recruitment ecology, as the combination of mobility and trophic traits could influence habitat selection and use by recruits and post‐recruits (see Booth and Wellington 1998; Brandl et al. 2015; Farmer and Ault 2011; Félix‐Hackradt et al. 2014; Giffin et al. 2019; Komyakova et al. 2019). For a complete list of species with their respective trophic and mobility traits, refer to Table A2.

### 2.3 Data Analysis

**FIGURE A1:**
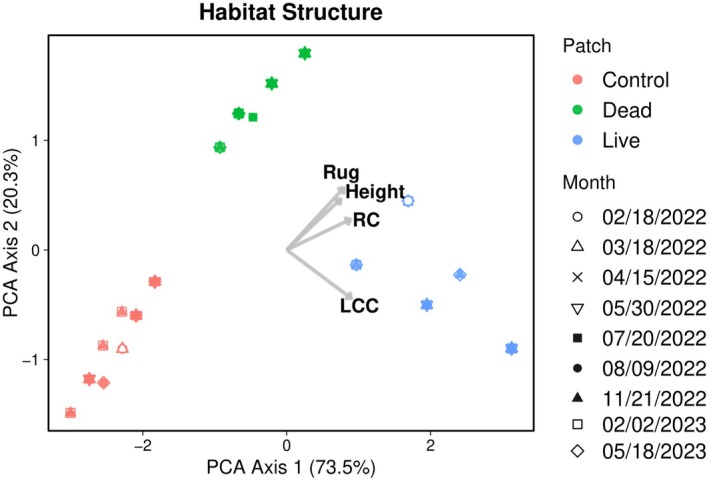
Biplot from the Principal Component Analysis of the physical structure in live, dead, and control patches. Height, average height of habitat architecture in cm; LCC, Live coral cover; RC, number of refuge categories; Rug, visual rank of the rugosity. See Table A1 for definitions.

Differences between live, dead, and control patches were visualized in a PCA based on the rank score from habitat structure variables (Figure A1). To evaluate differences in the total abundance and species richness of recruits and post‐recruits among patches, we fitted generalized linear mixed models (GLMMs) with a negative binomial error distribution (log link). Patch (control, dead, live) was included as a fixed factor and sampling date as a random intercept to account for repeated measures. Model assumptions were evaluated using simulation‐based residual diagnostics. Pairwise comparisons among patches were performed using Tukey‐adjusted estimated marginal means. We then plotted smoothed conditional means for the abundance and richness of the whole assemblage. We treated each patch monitored in a field survey as a sample unit—i.e., 12 sample units per survey.

**FIGURE A2:**
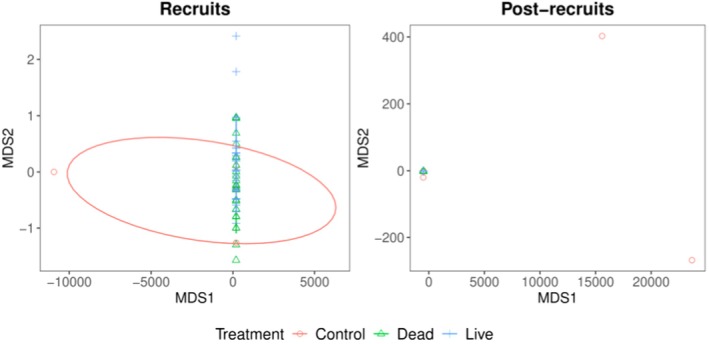
nMDS results displaying the original Bray–Curtis similarity from raw abundance‐count data for recruit and post‐recruit reef fish assemblages in the live dead, and control patches. This figure shows the over‐dispersion caused by one sample in recruit and three samples in post‐recruit assemblages. Ellipses indicate the 95% confidence interval. Stress < 0.001.

To compare differences in fish assemblages across patches, we first excluded all samples in which no fish were observed. We applied Hellinger transformation to normalize the data and then computed two Bray–Curtis dissimilarity matrices, one for recruits and one for post‐recruits. Then, two non‐metric multidimensional scaling (nMDS) ordinations were produced to visualize the multivariate dispersion of recruit and post‐recruit assemblages across treatments and controls. For the nMDS, we removed one sample out of 57 from recruit assemblages and three samples out of 83 from the post‐recruit assemblages as these were outliers from the control patcharea, with unusually high dissimilarity values that otherwise distorted the ordination (Figure A2).

To test for multivariate differences in fish assemblage structure between patches, we further performed a stratified permutational analysis of variance (PERMANOVA) with 999 permutations based on the same similarity matrix. We stratified the permutations in the PERMANOVA by month to account for repeated measures, allowing permutations within months but not between months. The experimental patches were used as the explanatory variables. Significant differences between patches were subsequently examined using pairwise comparisons. We could only run PERMANOVA for two out of five trophic groups in recruits and five out of nine groups in post‐recruits, due to low abundances.

To examine patches' effects on the mobility and trophic traits of associated fish assemblages, we modeled recruit and post‐recruit abundances by fitting GLMMs with post hoc pairwise comparisons following the same steps described above. We used patch and functional traits as fixed factors with interactions, and date as a random factor. Later we visualized the results with bar plots to display fish abundance per trophic group and mobility in all sampling months.

All data analyses were conducted using R software version 4.3.3 (R Core Team 2024). Dunn's test was performed using the *FSA* package version 0.9.6 (Ogle et al. 2025). The package *ape* version 5.8–1 (Paradis and Schliep 2019) was used to run the PCA. The *glmmTMB* package version 1.1.12 (McGillycuddy et al. 2025) was utilized to fit the GLMMs, and the *emmeans* package version 1.11.2–8 (Lenth 2023) for the pairwise comparisons. Simulation‐based residual diagnostics were obtained using the *DHARMa* package version 0.4.7 (Hartig 2024). We employed *vegan* version 2.6–2 (Oksanen et al. 2024) for data transformation, nMDS and PERMANOVA analyses, and used ‘pairwiseAdonis2’ for the pairwise PERMANOVAs (Martinez Arbizu 2020).

## 3 Results

**FIGURE A3:**
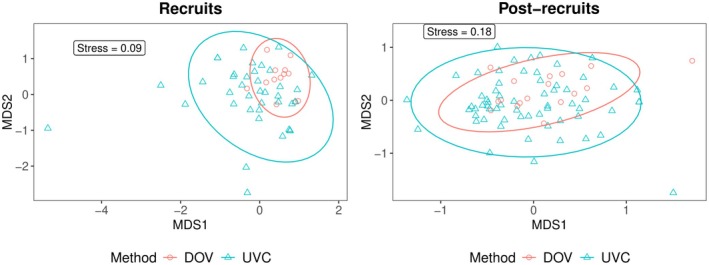
nMDS results comparing the results from DOV vs. UVC methods in the live coral dead coral, and control patches control area based on Bray‐Curtis distance matrices from the count data. Ellipses indicate a 95% confidence interval.

**FIGURE A4:**
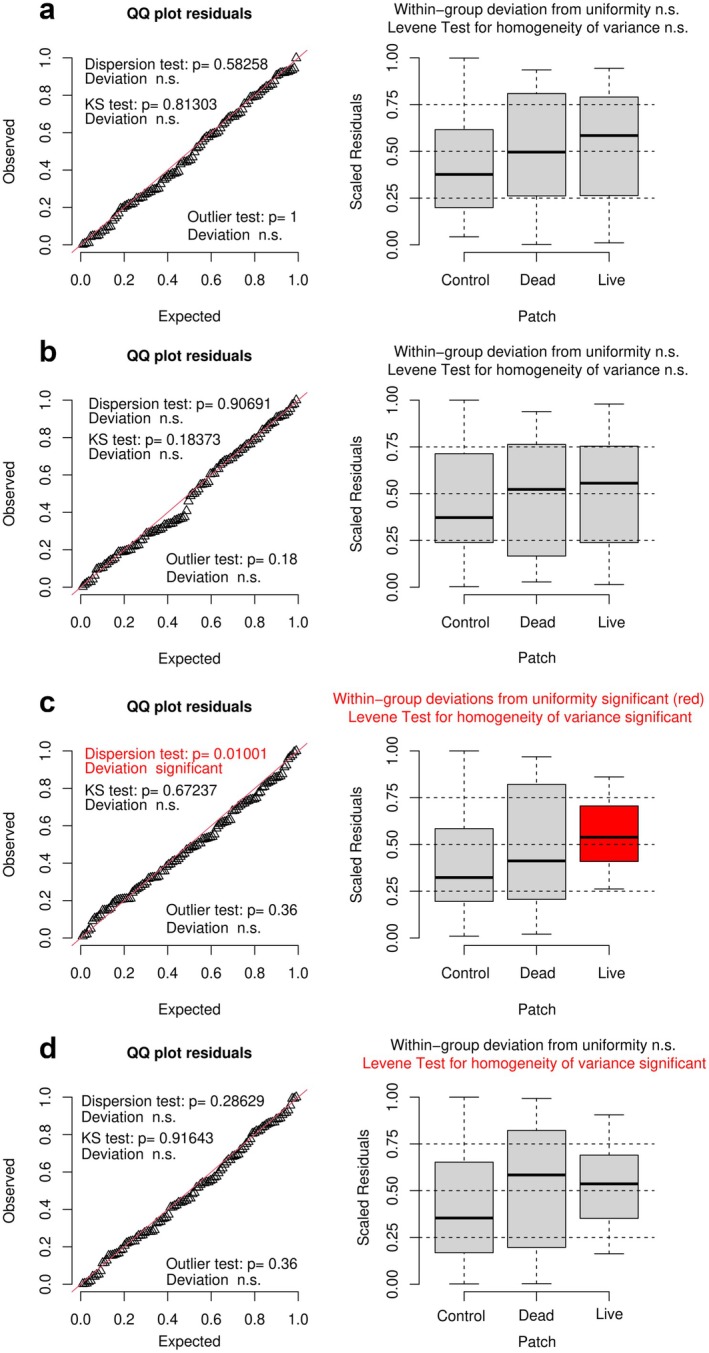
Residual diagnostics for GLMMs for the comparison of fish assemblages between patches. Residual diagnostics of (a) recruit abundance, (b) recruit richness, (c) post‐recruit abundance and (d) post‐recruit richness. Overdispersion was detected in post‐recruit richness. Significant heterogeneity of variances was detected in post‐recruit abundance and richness. Each dot represents a sampling unit from a single patch.

**FIGURE A5:**
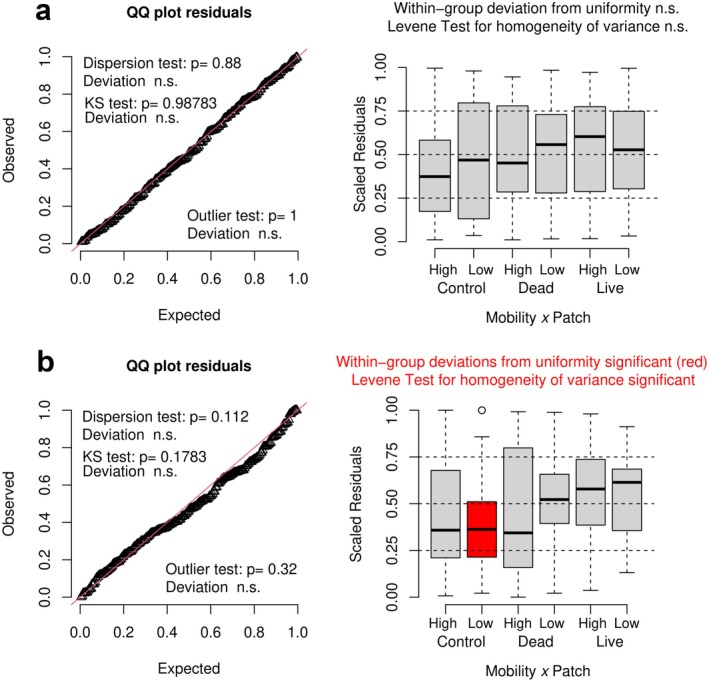
Residual diagnostics for GLMMs for the comparison of fish assemblages between patches according to high and low mobility traits. Residual diagnostics of (a) recruit assemblages, and (b) post‐recruit assemblages. Residuals followed a normal distribution with no overdispersion and outliers. Significant heterogeneity of variances was detected in post‐recruit assemblages. Each dot represents a sampling unit from a single patch.

**FIGURE A6:**
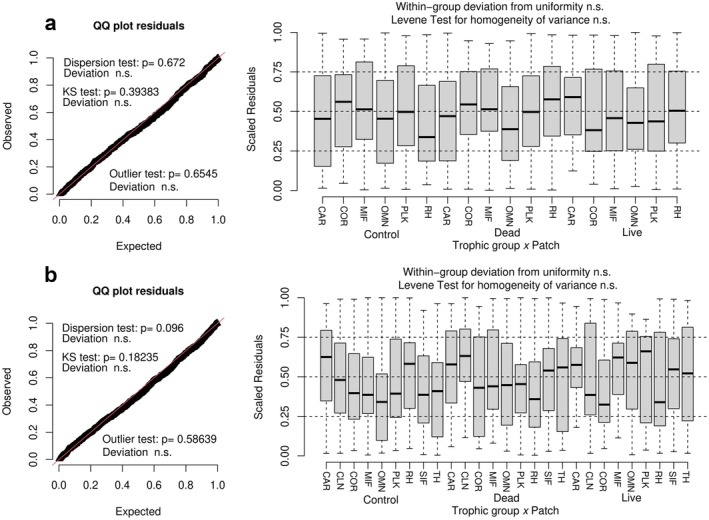
Residual diagnostics for GLMMs for the comparison of fish assemblages between patches according to trophic group. Trophic groups: (CAR, carnivore; CLN, cleaner; COR, corallivore; MIF, motile invertebrate feeder; OMN, omnivore; PLK, planktivore; RH, roving herbivore; SIF, sessile invertebrate feeder; TH, territorial herbivore). Residual diagnostics of (a) recruit assemblages, and (b) post‐recruit assemblages. Residuals followed a normal distribution with no overdispersion and outliers. No significant heterogeneity of variances was detected. Each dot represents a sampling unit from a single patch.

The PCA revealed that both live and dead coral patches were primarily associated with greater height and rugosity, whereas control patches were positioned in the opposite direction along both ordination axes. Differences between live and dead coral patches were mainly driven by the number of refuge categories (Figure A1). No significant differences were found between the abundance and richness of fishes recorded by UVC or DOV methods (Figure A3; Tables A3 and A4). Additionally, the simulation‐based residual diagnostics indicated acceptable model fit in most of our GLMMs (no substantial deviations in dispersion or residual patterns) (see Figures A4, A5, A6).

### 3.1 Effects of Patches Across Life Stages

**FIGURE 2:**
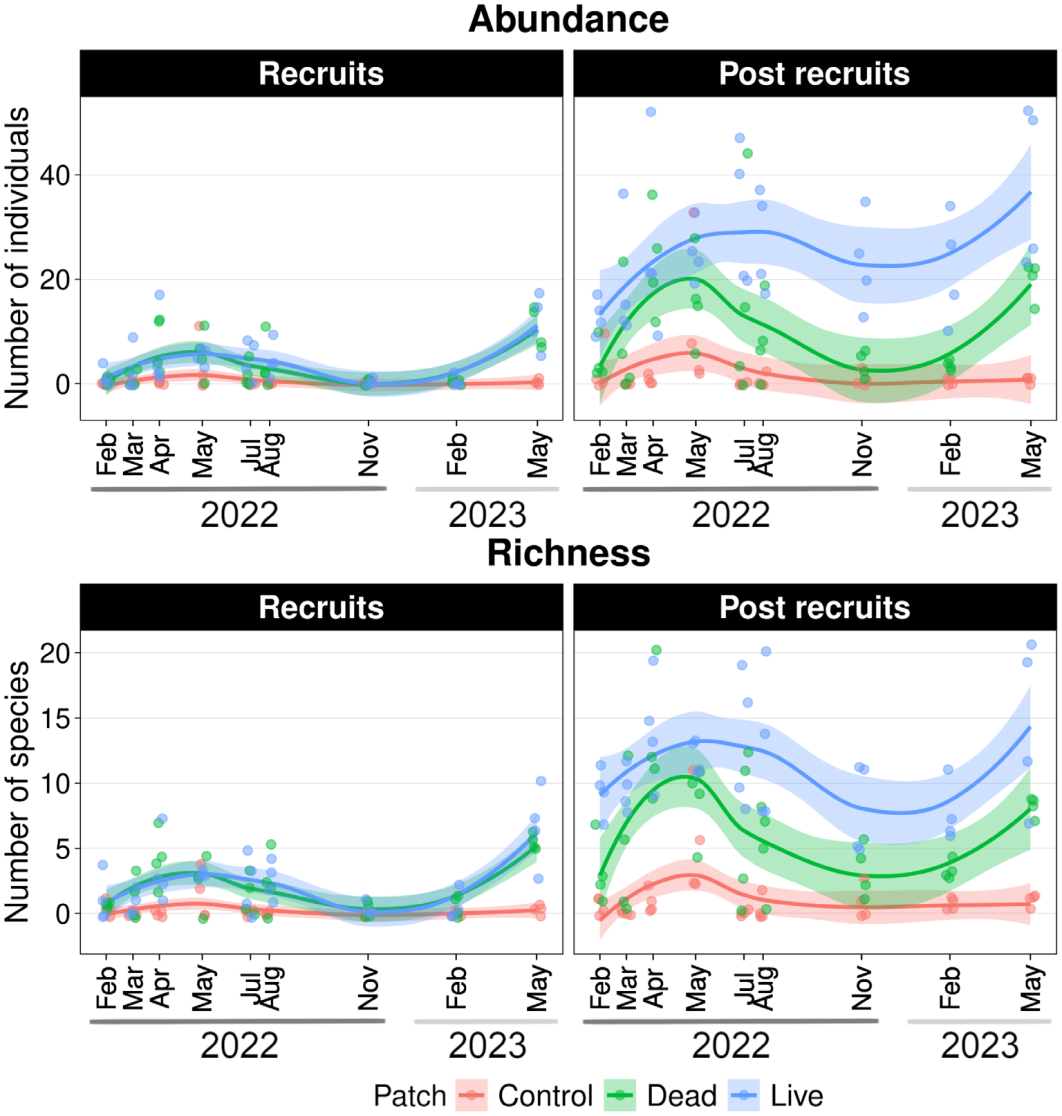
Abundance and species richness of recruit and post‐recruit reef fish assemblages in live coral, dead coral, and control patches over the one‐year experimental period. Points represent individual samples, and the lines and shaded areas represent the smoothed conditional means with 95% confidence intervals. Ticks at the x‐axis are scaled by date.

**FIGURE 3:**
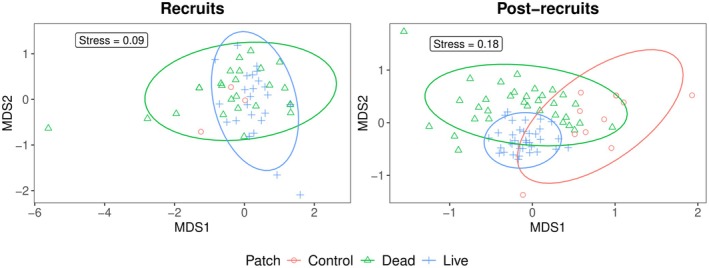
nMDS results displaying the similarity of recruit and post‐recruit reef fish assemblages in live coral, dead coral, and control patches, based on Bray‐Curtis distance matrices from Hellinger transformed data. Ellipses represent a 95% confidence interval. Due to high collinearity between recruits' data points in the control patches, the ellipse could not be calculated.

**FIGURE A7:**
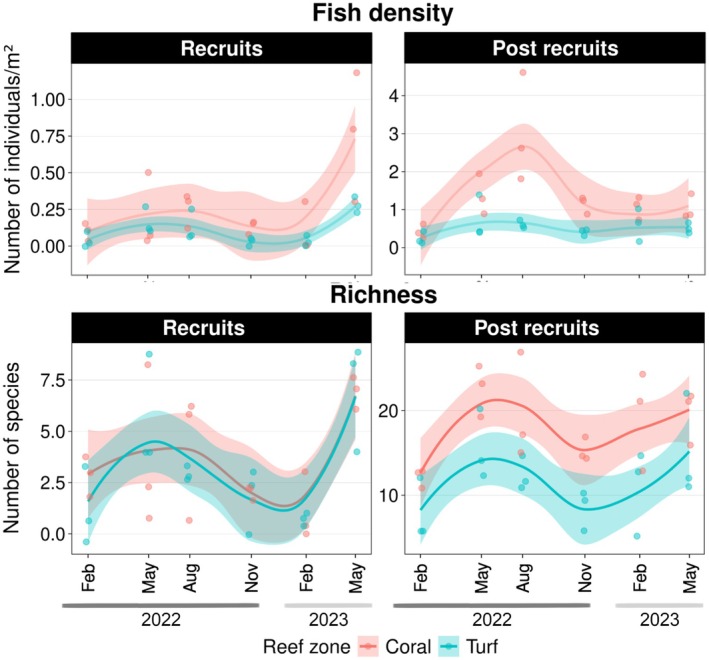
Abundance and richness of recruits and post‐recruits per transect in the turf and the coral zones of Shanfu reef. Points represent individual samples, lines and the shaded areas represent the smoothed conditional means with the respective 95% confidence intervals.

Recruit and post‐recruit assemblages varied seasonally (Figure 2) following local dynamics (Figure A7). Recruits were significantly more abundant and species‐rich on live and dead patches compared to control patches (abundance: *z* = 5.56 and 6.01, *p* < 0.001; richness: *z* = 5.89 and 6.10, *p* < 0.001), whereas live and dead patches did not differ significantly from each other (Tables A5 and A6). The nMDS showed substantial overlap between recruit assemblage composition in live and dead coral patches (Figure 3), while the PERMANOVA indicated marginal differences between these assemblages (*R* ^2^ = 0.04, *p* < 0.05; Tables A7 and A8). In general, recruitment densities per m^2^ were higher in the experimental treatments than in the adjacent turf and coral zones (see Figure A7).

Post‐recruits also showed markedly higher abundance and richness on live and dead patches compared to control patches (abundance: *z* = 7.38 and 10.75, *p* < 0.001; richness: z = 8.86 and 12.21, *p* < 0.001), and were significantly more abundant and species‐rich on live than dead patches (abundance: *z* = −4.24, *p* = 0.001; richness: *z* = −4.94, *p* < 0.001). Results from PERMANOVA (*R* ^2^ = 0.16, *p* < 0.01; Tables A7 and A8) indicated significant differences in assemblage structure among live, dead, and control patches. Live coral patches differed from dead and control patches to a greater extent (PERMANOVA, *R* ^2^ = 0.14 and 0.17, respectively, *p* = 0.001), whereas weaker differences were observed between dead and control patches (*R* ^2^ = 0.05, *p* = 0.001) (Figure 3 and Table A8).

### 3.2 Influence of Patches on Species Traits

**FIGURE 4:**
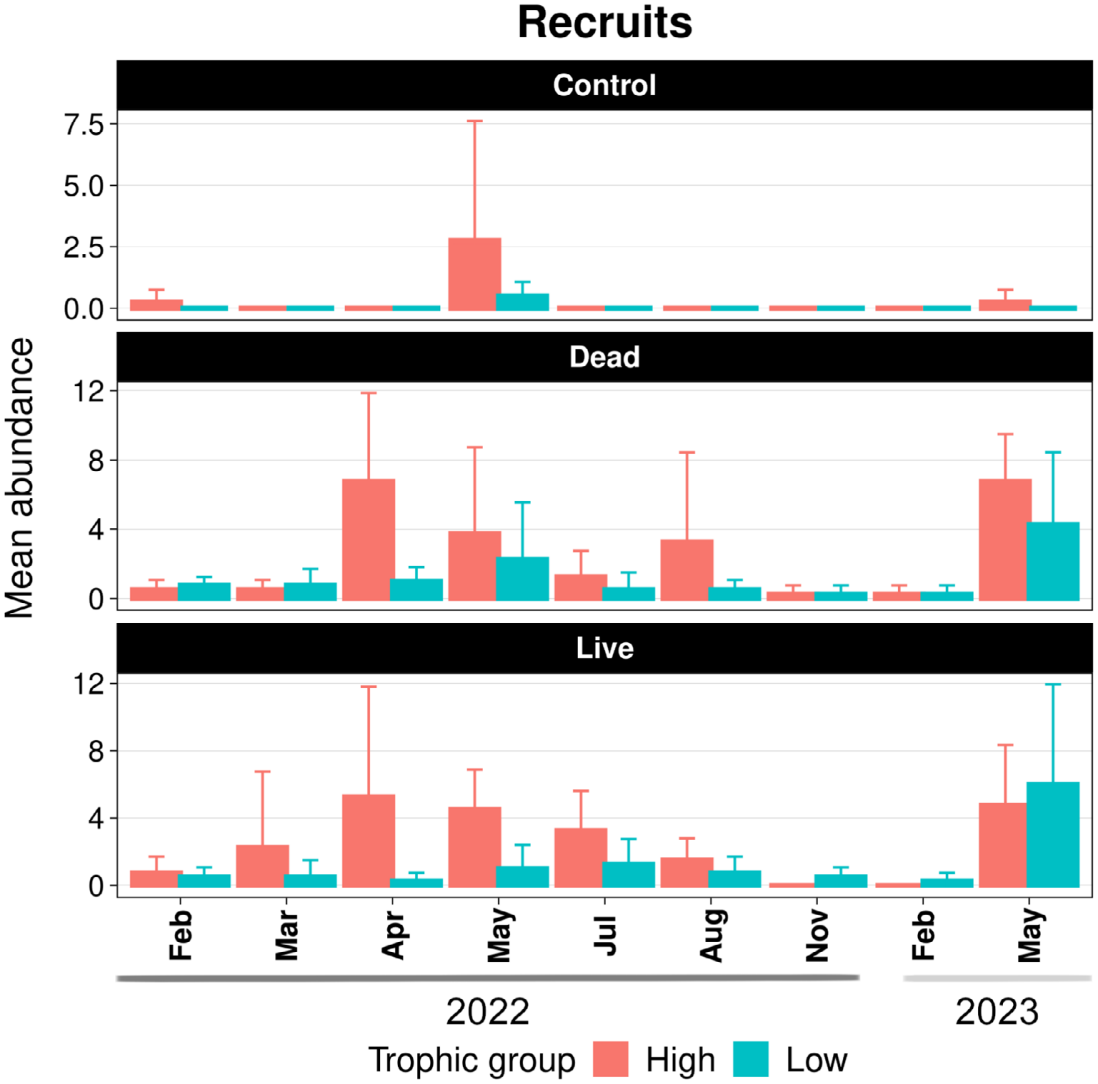
Mean abundance of recruits per month according to different mobility traits. Error bars indicate the standard deviation.

**FIGURE 5:**
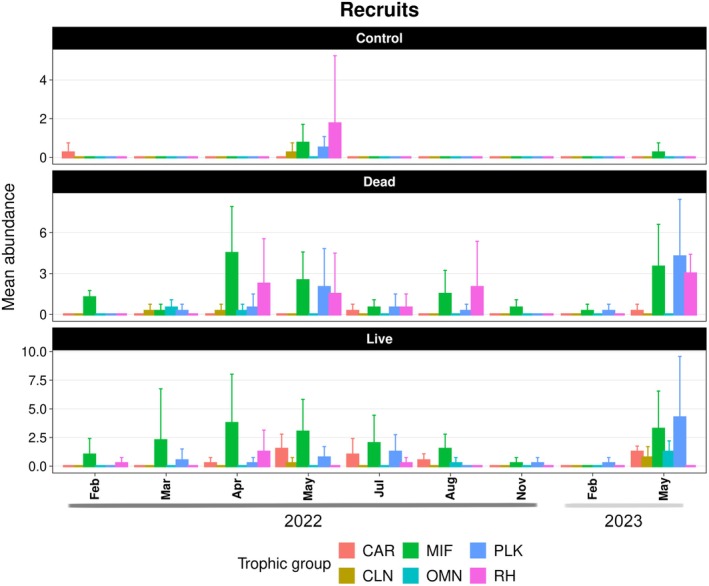
Mean abundance of recruits and post‐recruits per month according to different trophic groups. Error bars indicate the standard deviation. CAR, carnivore; CLN, cleaner; COR, corallivore; MIF, motile invertebrate feeder; OMN, omnivore; PLK, planktivore; RH, roving herbivore.

Throughout the experimental period, live and dead coral patches supported similar abundances of both high‐ and low‐mobility recruit species (Figure 4; Tables A9 and A10). Trophic groups also showed comparable abundances between the two treatments, with no significant differences detected for motile invertebrate feeders or planktivores (Figure 5; Tables A11 and A12). However, distinct patterns emerged in other groups: live coral patches supported significantly more carnivores (*z* = −2.62, *p* = 0.02), while dead coral patches harbored higher numbers of roving herbivores (*z* = 2.81, *p* = 0.01; Tables A11 and A12).

**FIGURE 6:**
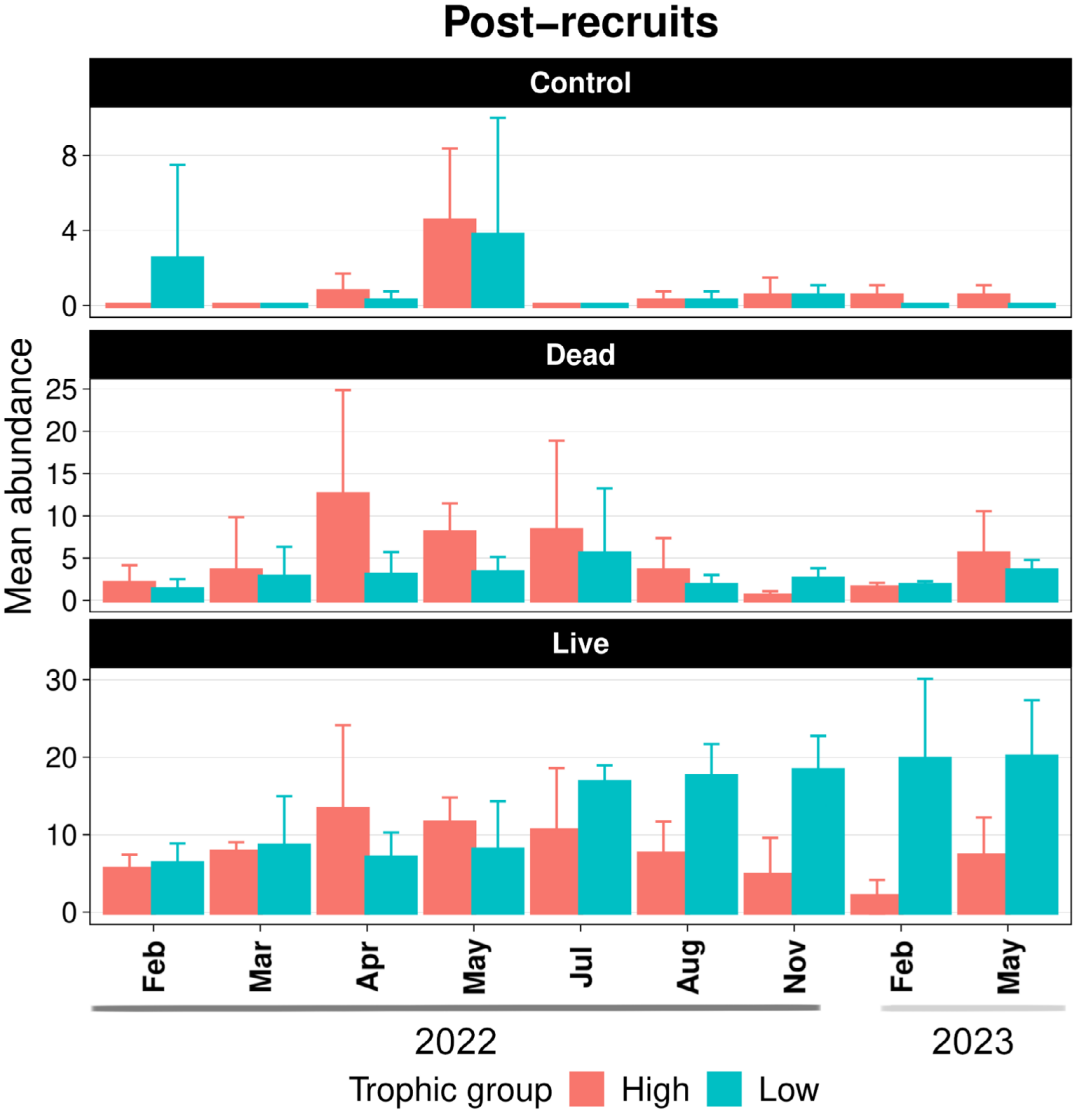
Mean abundance of post‐recruits per month according to different mobility traits. Error bars indicate the standard deviation.

**FIGURE 7:**
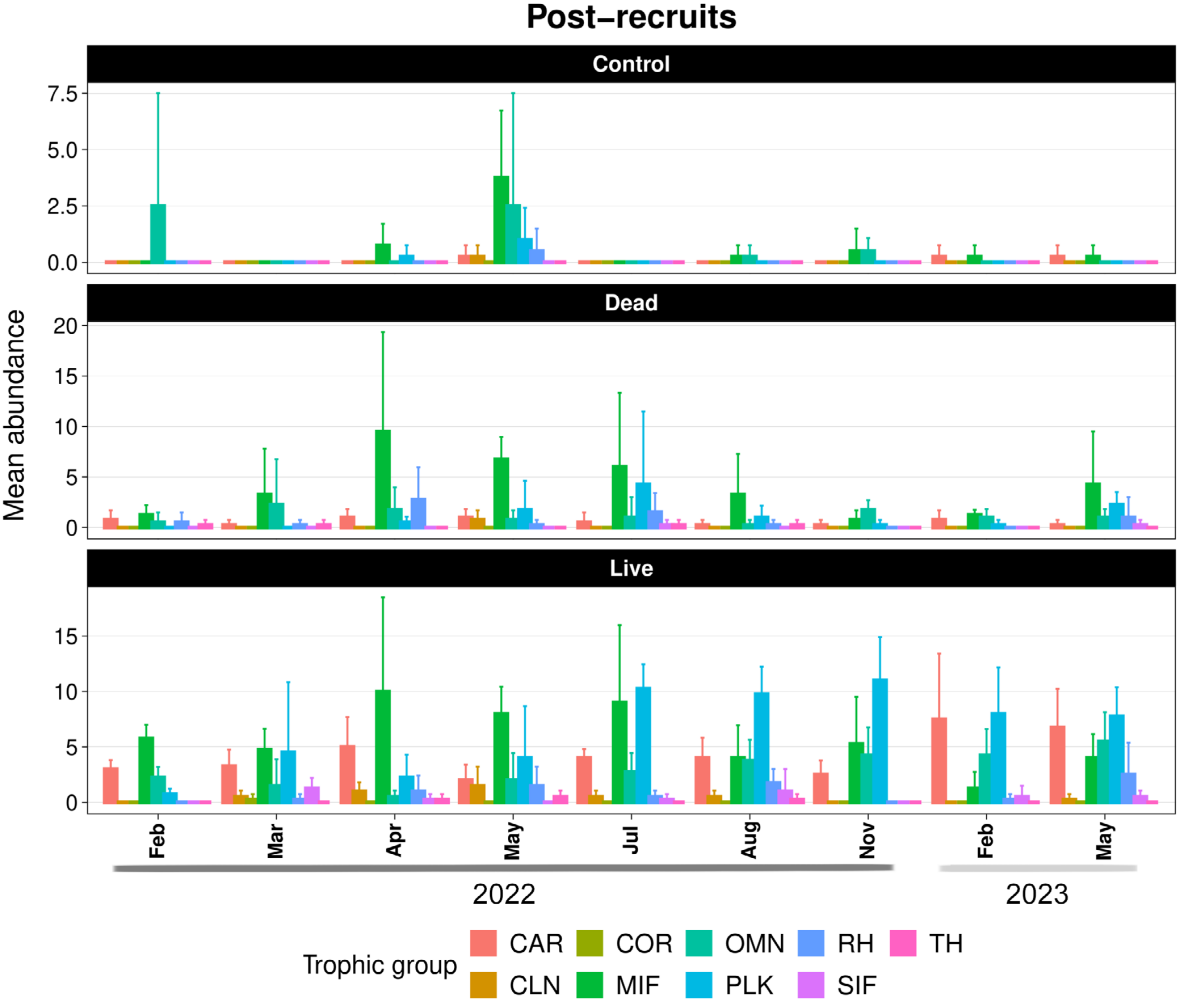
Mean abundance of post‐recruits per month according to different trophic groups. Error bars indicate the standard deviation. CAR, carnivore; CLN, cleaner; COR, corallivore; MIF, motile invertebrate feeder; OMN, omnivore; PLK, planktivore; RH, roving herbivore; SIF, sessile invertebrate feeder; TH, territorial herbivore.

For post‐recruits, live and dead coral patches exhibited similar seasonal patterns in the abundance of high‐mobility species, both following trends of motile invertebrate feeders. In contrast, low‐mobility species in both patches followed the seasonal dynamics of planktivores, with live patches consistently supporting higher abundances (*z* = −7.11, *p* < 0.001) (Figures 6 and 7; Tables A9 and A10). Although high‐mobility species showed no significant difference between live and dead patches, planktivores were significantly more abundant in live patches (*z* = −6.48, *p* < 0.001). Over time, live patches transitioned from being dominated by mobile species—especially motile invertebrate feeders—to assemblages increasingly composed of low‐mobility planktivores. Additionally, live patches had significantly higher abundances of carnivores (*z* = −6.29, *p* < 0.001) and omnivores (*z* = −3.33, *p* < 0.001) compared to dead patches (see Tables A13 and A14).

## 4 Discussion

Recruit assemblages showed similarities between live and dead coral patches, both showing greater abundance and richness than the control patches. The assemblages in live and dead patches were dominated by only two trophic groups and exhibited similar species composition between patches, underscoring the potential of dead corals as temporary shelters in degraded habitats. On the other hand, post‐recruits displayed stronger habitat‐specific responses. Live coral patches supported higher abundance, species richness, and more trophic groups, indicating that mature individuals might benefit from the structural stability and increased resources provided by live corals.

Spatial distribution patterns of recruits are strongly influenced by the availability of shelter, primarily due to predation risk (Almany 2004b; Steele 1999). The low structural complexity characteristic of our control patches offers little protection, leading to lower recruit abundance and richness (Booth and Beretta 1994; Félix‐Hackradt 2013) while both live and dead coral patches support similar recruit assemblages. Conversely, post‐recruit abundances showed marked differences between live and dead habitats. These differences suggest that shelter holds a higher influence on recruit habitat choice in our coral patches than post‐recruit density, a pattern consistent with previous observations (Almany 2004a). The relatively high complexity and structural protection possibly reduced young fish mortality (Almany 2004b; Cabaitan et al. 2008; Cheminée et al. 2016), contributing to increased fish biomass (Beese et al. 2023). This highlights the potential role of live and dead coral patches in the recovery of reef fish assemblages.

The similarity between recruit assemblages in live and dead coral patches was partially driven by highly mobile species of invertebrate feeders. These fishes typically utilize several reef habitats for foraging and shelter (Lecchini and Galzin 2005), including both live and dead corals (Almany 2004a; Ferreira et al. 2015; Giffin et al. 2019; Johansson et al. 2012). While live corals support abundant associated fauna (Patton 1994), dead corals also retain ecological importance, sustaining diverse assemblages of small invertebrates even after coral death (Head et al. 2015). This makes both habitats viable foraging grounds for motile invertebrate feeders (Bellwood 1988; Chen 2002; Choat 1991; Kimirei et al. 2013; Lecchini and Galzin 2005). Thus, for highly mobile species, dead corals could represent an important alternative to live corals (Giffin et al. 2019; Suzuki et al. 2018).

The added habitat complexity created by dead and live corals helps reduce competition, predation risks, and the impact of environmental stressors (Almany 2004b). Migrants from the surrounding area are often attracted to the benefits of complex habitats, leading to an aggregation effect (see Acosta and Robertson 2002; Schroeder 1987), leading to a higher fish density in treatment patches compared to the turf and coral zones, which feature a patchy distribution of complex and flat areas. Over time, this aggregation effect was more pronounced in live coral patches, which constantly supported greater abundance, species richness, and number of trophic groups. This likely reflects the greater structural stability and persistence in live coral patches, which maintain longer‐lasting habitat complexity, while dead coral colonies gradually erode with time (Cheung et al. 2021; Darling et al. 2017).

Beyond structural benefits, live corals provide energetic and ecological resources, attracting both directly and indirectly associated species (Coker et al. 2013; Komyakova 2018; Quimbayo et al. 2019; Yap et al. 1994). This includes mobile species with large home ranges, like the motile invertebrate feeders *Stethojulis trilineata* and *Thalassoma quinquevitatum*, which initially dominated live coral patches due to their mobile capacity and attraction toward coral‐associated invertebrates (Chapman and Kramer 2000; Ferreira et al. 2015; Suzuki et al. 2018). Over time, however, live coral patches became increasingly dominated by planktivores such as *Dascyllus reticulatus* , which usually form large shoals (Floeter et al. 2006; Holbrook et al. 2000; Russ et al. 2020). These sedentary groups benefit from structural stability and are often found in association with live corals (Booth and Beretta 2002; Cabaitan et al. 2008; Coker et al. 2012; Komyakova et al. 2013). Those patches consequently attracted carnivores and omnivores that forage on the associated fauna and surrounding substrate (Kramer et al. 2015; Osuka et al. 2022; Stier and Leray 2014). Hence, while relatively few reef fish species are strongly associated with corals (Siqueira et al. 2023), live corals attract a diverse array of species with varied traits, supporting a larger number of trophic groups than dead corals or turf substrates (see Coker et al. 2013; Mouillot et al. 2014; Munday 2004; Pratchett et al. 2011; Wilson et al. 2008).

The similar spatial and temporal trends observed in recruits and post‐recruits of highly mobile species suggest limited ontogenetic habitat shifts, contrasting with previous findings (Giffin et al. 2019). Conversely, sedentary‐planktivorous species exhibited shifts toward more specialized habitat use in post‐recruit stages. Ontogenetic transitions often reflect changes from generalist recruits to specialized pre‐adults and adults (Feary et al. 2007; Lirman 1994). The varying patterns in ontogenetic shifts across trophic groups found here and the discrepancy with other studies highlight an important gap in our understanding of life‐stage‐specific habitat use. Particularly, more research is needed on the functional traits of early life stages, as most trait‐based studies still focus primarily on adults.

While our study was limited to 1 m^2^ patches composed of two *Pocillopora* spp. in a degraded reef zone, the findings provide valuable insights into habitat function across fish life stages. Despite spatial and compositional constraints, our design offers a controlled foundation for future research into habitat‐specific recruitment at broader scales. For instance, fast‐growing branching corals, such as *Pocillopora* sp., are linked to rapid coral cover recovery in the Indo‐Pacific (Gilmour et al. 2013; Mulla et al. 2024). As shown here, even small patches of these corals support diverse fish assemblages spanning multiple trophic groups. Future studies should further explore how coral diversity, species identity, and patch size interact to shape recruit and post‐recruit assemblages under varying reef conditions.

## 5 Conclusions

Our findings indicate that dead branching coral patches are not “graveyards”; rather, their retained complexity may serve as transitional shelters, helping to buffer the negative impacts of coral mortality on fish assemblages (Emslie et al. 2014; Morais et al. 2022). However, live corals remain irreplaceable for supporting functionally diverse assemblages across multiple trophic levels, a crucial component of ecosystem health (Graham et al. 2011; MacNeil et al. 2015). The patchy habitat configurations used here—often employed in restoration projects—could be important attractors for recruits and post‐recruits, supporting diverse functional traits, which ensures a link between bottom‐up and top‐down processes (Beese et al. 2023). Future studies at larger spatial and temporal scales could build on these findings to clarify how fish from different life stages and functional groups respond to live, dead, and even coral rubble habitats, a commonly overlooked substrate. Integrating habitat‐specific strategies—safeguarding live corals for their high productivity and functional importance, while also protecting dead corals from further fragmentation—could meaningfully enhance the resilience of coral reef fish assemblages in degraded ecosystems.

## Author Contributions

**Felipe M. G. Mattos:** conceptualization (equal), data curation (lead), formal analysis (lead), investigation (lead), methodology (equal), visualization (lead), writing – original draft (lead), writing – review and editing (lead). **Aziz J. Mulla:** investigation (supporting), writing – original draft (equal), writing – review and editing (equal). **Vianney Denis:** formal analysis (supporting), writing – original draft (equal), writing – review and editing (equal). **Che‐Hung Lin:** investigation (supporting), methodology (supporting), writing – review and editing (supporting). **Tzu‐Hao Lin:** formal analysis (supporting), supervision (equal), visualization (supporting), writing – original draft (equal), writing – review and editing (equal). **Yoko Nozawa:** conceptualization (equal), formal analysis (supporting), funding acquisition (lead), investigation (supporting), methodology (equal), project administration (lead), resources (lead), supervision (equal), visualization (supporting), writing – original draft (equal), writing – review and editing (equal).

## Conflicts of Interest

The authors declare no conflicts of interest.

## Data Availability

The data supporting the findings of this study are openly available in the depositar repository (https://pid.depositar.io/ark:37281/k5f4j959b).

## Appendix A

**TABLE A1:** Definitions and explanations of the factors and ranks used to measure the physical structure of the patches.

	Rank 1	Rank 2	Rank 3	Rank 4	Rank 5
Rugosity (visual topographic estimate of the substratum in each patch)	Mostly flat (e.g., sandy matrix)	Flat with bumps but no holes	Shallow holes and peaks	Mostly vertical but shallow holes, gaps and short peaks	Vertical and horizontal holes, gaps, peaks and a maze‐like structure
Height (average height of habitat architecture in cm)	0–9	10–19	20–39	40–79	> 80
Refuge size categories (i.e., how many different sizes the available refuges have). Categories: 1–5, 6–15, 16–30, 31–50 and > 50 cm.	0–1	2	3	4	5
Live coral cover (%)	0–19	20–39	40–59	60–79	80–100

**TABLE A2:** List of reef fish species recorded in experimental patches (Control, Dead coral, and Live coral). For each species we indicate whether it was observed as a recruit (Rec), post‐recruit (PR), or both in each patch. Trophic groups: (CAR, carnivore; CLN, cleaner; COR, corallivore; MIF, motile invertebrate feeder; OMN, omnivore; PLK, planktivore; RH, roving herbivore; SIF, sessile invertebrate feeder; TH, territorial herbivore). Mobility (High or Low). Mean settlement size is reported in millimeters (mm) only for species seen as recruits. Sources of the settlement sizes were cited in subsection 2.2.1 and can be obtained in the supporting dataset.

Species	Control	Dead	Live	Trophic group	Mobility	Settlement size (mm)
*Acanthurus japonicus*		PR		RH	High	—
*Acanthurus leucocheilus*			PR	RH	High	—
*Anampses caeruleopunctatus*		PR		MIF	High	—
*Anampses geographicus*	PR	Rec PR	PR	MIF	High	9
*Anampses melanurus*		Rec	Rec	MIF	High	9
*Anampses twistii*		PR		MIF	High	—
*Calotomus carolinus*		Rec PR	PR	RH	High	12
*Canthigaster valentini*		PR	PR	SIF	Low	—
*Centropyge vrolikii*		PR	PR	TH	Low	—
*Cephalopholis urodeta*		PR	PR	CAR	Low	—
*Chaetodon argentatus*		PR		OMN	High	—
*Chaetodon citrinellus*			PR	OMN	High	—
*Chaetodon kleinii*		PR		OMN	High	—
*Chaetodon lunulatus*			PR	COR	High	—
*Chaetodon rafflesi*		PR	PR	SIF	High	—
*Chaetodon trifascialis*		PR	PR	COR	High	—
*Cheilinus oxycephalus*			PR	CAR	Low	—
*Cheilinus trilobatus*		PR	PR	CAR	High	—
*Chlorurus japanensis*		PR	PR	RH	High	—
*Chlorurus microrhinos*		Rec PR	Rec PR	RH	High	10
*Chlorurus spilurus*	Rec PR	Rec PR	Rec PR	RH	High	10
*Chromis weberi*		PR		PLK	Low	—
*Cirrhilabrus cyanopleura*		PR	PR	PLK	Low	—
*Cirrhilabrus exquisitus*	PR	Rec PR		PLK	Low	7.8
*Cirrhilabrus melanomarginatus*		Rec PR	Rec PR	PLK	Low	7.8
*Cirrhitichthys aprinus*			PR	CAR	Low	—
*Cirrhitichthys falco*		PR	PR	CAR	Low	—
*Cirrhitichthys oxycephalus*		PR	PR	CAR	Low	—
*Coris aygula*		PR		MIF	High	—
*Coris caudimacula*	PR			MIF	High	—
*Coris gaimard*	Rec	Rec	Rec PR	MIF	High	11
*Ctenochaetus binotatus*			PR	RH	High	—
*Ctenochaetus striatus*		PR	PR	RH	High	—
*Dascyllus reticulatus*			Rec PR	PLK	Low	11.1
*Dascyllus trimaculatus*		PR	Rec PR	PLK	Low	11.1
*Dendrochirus zebra*		PR	PR	CAR	Low	—
*Gomphosus varius*		Rec PR	Rec PR	CAR	High	15
*Grammistes sexlineatus*		PR		CAR	High	—
*Gymnothorax meleagris*		PR		CAR	High	—
*Halichoeres biocellatus*		Rec PR	PR	MIF	High	10
*Halichoeres chrysus*		PR	PR	MIF	High	—
*Halichoeres hortulanus*		Rec PR	Rec PR	MIF	High	10
*Halichoeres margaritaceus*	PR	PR		MIF	High	—
*Halichoeres nebulosus*	PR	PR	PR	MIF	High	—
*Hemigymnus fasciatus*		PR	PR	MIF	High	—
*Hologymnosus doliatus*	PR			MIF	High	—
*Labroides bicolor*			PR	CLN	High	—
*Labroides dimidiatus*	Rec PR	Rec PR	Rec PR	CLN	High	12.6
*Macropharyngodon meleagris*	Rec PR	Rec PR	Rec PR	MIF	High	9
*Macropharyngodon negrosensis*		Rec PR	PR	MIF	High	9
*Meiacanthus grammistes*	PR		PR	PLK	Low	—
*Ostracion meleagris*			PR	OMN	High	—
*Oxycheilinus bimaculatus*		PR	PR	MIF	High	—
*Oxycheilinus rhodochrous*		Rec		MIF	High	10
*Oxycheilinus unifasciatus*		Rec PR	PR	MIF	High	10
*Paracirrhites arcatus*		PR	PR	CAR	Low	—
*Paracirrhites forsteri*			PR	CAR	Low	—
*Parapercis clathrata*	PR	PR		CAR	High	—
*Parapercis hexophthalma*	PR			CAR	High	—
*Parupeneus barberinus*			Rec PR	MIF	High	37
*Parupeneus multifasciatus*		Rec		MIF	High	12.6
*Plectorhinchus picus*			PR	CAR	High	—
*Plectorhinchus vittatus*	Rec	PR	PR	CAR	High	9.6
*Plectroglyphidodon dickii*			Rec PR	OMN	Low	11.3
*Plectroglyphidodon lacrymatus*		PR	PR	OMN	Low	—
*Pomacentrus bankanensis*		Rec PR	PR	OMN	Low	14.4
*Pomacentrus chrysurus*		Rec PR		OMN	Low	13.3
*Pomacentrus coelestis*	PR	PR	PR	OMN	Low	—
*Pomacentrus vaiuli*		PR	PR	OMN	Low	—
*Pseudanthias squamipinnis*		Rec	Rec	PLK	Low	1
*Pseudocheilinus evanidus*		PR		MIF	Low	—
*Pseudocheilinus hexataenia*		Rec PR	Rec PR	MIF	Low	5
*Pterois antennata*			PR	MIF	Low	—
*Pterois radiata*			PR	MIF	Low	—
*Pterois volitans*			PR	CAR	Low	—
*Pycnochromis margaritifer*	Rec PR	Rec PR	Rec PR	PLK	Low	14.5
*Pycnochromis vanderbilti*		Rec PR	Rec	PLK	Low	14.5
*Scarus forsteni*		Rec	Rec PR	RH	High	15
*Scarus globiceps*			PR	RH	High	—
*Scarus hypselopterus*			PR	RH	High	—
*Scarus psittacus*		Rec PR	PR	RH	High	12
*Scorpaenopsis papuensis*			PR	CAR	Low	—
*Sebastapistes cyanostigma*	PR		PR	CAR	Low	—
*Stegastes nigricans*			PR	TH	Low	—
*Stethojulis bandanensis*	PR	PR	PR	MIF	High	—
*Stethojulis trilineata*		Rec PR	Rec PR	MIF	High	7
*Thalassoma amblycephalum*		Rec PR	Rec PR	MIF	High	14
*Thalassoma hardwicke*	PR	Rec PR	Rec PR	MIF	High	14
*Thalassoma jansenii*		PR	PR	MIF	High	—
*Thalassoma lunare*			Rec	MIF	High	11
*Thalassoma lutecens*	PR	Rec PR	PR	MIF	High	12.5
*Thalassoma purpureum*			PR	MIF	High	—
*Thalassoma quinquevittatum*	PR	Rec PR	Rec PR	MIF	High	14
*Zanclus cornutus*		PR	PR	MIF	High	—

**TABLE A3:** Results of the pairwise Dunn's test for the abundance and richness of recruits between months according to DOV and UVC methods. Stars indicate months when DOV was the collection method.

Month pair		*Z*	*p*.adj
*Recruit abundance*			
2022‐03‐18	*2022‐04‐15	−1.74	0.20
2022‐03‐18	2022‐05‐30	−2.37	0.07
*2022‐04‐15	2022‐05‐30	−0.62	0.66
2022‐03‐18	*2022‐07‐20	−0.53	0.67
*2022‐04‐15	*2022‐07‐20	1.21	0.39
2022‐05‐30	*2022‐07‐20	1.83	0.19
2022‐03‐18	2022‐08‐09	−1.15	0.41
2022‐04‐15	2022‐08‐09	0.59	0.65
2022‐05‐30	2022‐08‐09	1.21	0.43
*2022‐07‐20	2022‐08‐09	−0.62	0.64
*Recruit richness*			
2022‐03‐18	*2022‐04‐15	−2.03	0.14
2022‐03‐18	2022‐05‐30	−2.39	0.06
*2022‐04‐15	2022‐05‐30	−0.36	0.76
2022‐03‐18	*2022‐07‐20	−0.58	0.67
*2022‐04‐15	*2022‐07‐20	1.45	0.29
2022‐05‐30	*2022‐07‐20	1.81	0.18
2022‐03‐18	2022‐08‐09	−1.3	0.37
*2022‐04‐15	2022‐08‐09	0.73	0.62
2022‐05‐30	2022‐08‐09	1.09	0.47
*2022‐07‐20	2022‐08‐09	−0.72	0.61

**TABLE A4:** Results of the pairwise Dunn's test for the abundance and richness of post‐recruits between months according to DOV and UVC methods. Stars indicate months when DOV was the collection method.

Month pair		*Z*	*p*.adj
*Post‐recruit abundance*			
2022‐03‐18	*2022‐04‐15	−1.48	0.62
2022‐03‐18	2022‐05‐30	−2.23	0.92
*2022‐04‐15	2022‐05‐30	−0.75	0.86
2022‐03‐18	*2022‐07‐20	−0.82	0.83
*2022‐04‐15	*2022‐07‐20	0.67	0.91
2022‐05‐30	*2022‐07‐20	1.42	0.51
2022‐03‐18	2022‐08‐09	−0.65	0.84
*2022‐04‐15	2022‐08‐09	0.83	0.86
2022‐05‐30	2022‐08‐09	1.58	0.58
*2022‐07‐20	2022‐08‐09	0.16	0.92
*Post‐recruit richness*			
2022‐03‐18	*2022‐04‐15	−1.81	0.36
2022‐03‐18	2022‐05‐30	−2.12	0.41
*2022‐04‐15	2022‐05‐30	−0.31	0.94
2022‐03‐18	*2022‐07‐20	−0.55	0.88
*2022‐04‐15	*2022‐07‐20	1.26	0.53
2022‐05‐30	*2022‐07‐20	1.57	0.42
2022‐03‐18	2022‐08‐09	−0.36	0.96
*2022‐04‐15	2022‐08‐09	1.45	0.48
2022‐05‐30	2022‐08‐09	1.76	0.31
*2022‐07‐20	2022‐08‐09	0.19	0.96

**TABLE A5:** GLMM outputs for the comparison of recruit and post‐recruit abundances and richness among patch types. The estimated coefficients (log scale), standard errors, 95% confidence intervals, *z*‐values, and *p*‐values of fixed effects are presented. Significant differences (*p* < 0.05) are highlighted in bold.

	Estimate	SE	Lower limit	Upper limit	*z*	Pr (>|z|)
*Recruit abundance*						
Control (Intercept)	−1.52	0.52	−2.53	−0.5	−2.92	**< 0.001**
Dead	2.36	0.42	1.53	3.19	5.56	**< 0.001**
Live	2.56	0.43	1.73	3.4	6.01	**< 0.001**
*Recruit richness*						
Control (Intercept)	−1.8	0.45	−2.69	−0.91	−3.97	**< 0.001**
Dead	2.24	0.38	1.49	2.99	5.89	**< 0.001**
Live	2.31	0.38	1.57	3.06	6.1	**< 0.001**
*Post‐recruit abundance*						
Control (Intercept)	0.41	0.26	−0.1	0.91	1.58	0.11
Dead	1.94	0.26	1.42	2.45	7.38	**< 0.001**
Live	2.85	0.27	2.33	3.37	10.75	**< 0.001**
*Post‐recruit richness*						
Control (Intercept)	−0.05	0.21	−0.46	0.36	−0.24	0.81
Dead	1.81	0.2	1.41	2.21	8.86	**< 0.001**
Live	2.45	0.2	2.06	2.84	12.21	**< 0.001**

**TABLE A6:** Post hoc pairwise comparisons of recruit and post‐recruit abundances and richness among patches. Estimated marginal means contrasts are presented with log‐scale estimates, standard error, *z*‐ratio, and Tukey‐adjusted *p*‐values. Significant differences (*p* < 0.05) are highlighted in bold. Tests are based on Wald *z*‐statistics with asymptotic standard errors (df = ∞).

	Estimate	SE	*z*.ratio	*p*
*Recruit abundance*				
Control‐Dead	−2.36	0.42	−5.56	**< 0.001**
Control‐Live	−2.56	0.43	−6.01	**< 0.001**
Dead‐Live	−0.21	0.29	−0.72	0.75
*Recruit richness*				
Control‐Dead	−2.24	0.38	−5.89	**< 0.001**
Control‐Live	−2.31	0.38	−6.1	**< 0.001**
Dead‐Live	−0.07	0.18	−0.42	0.91
*Post‐recruit abundance*				
Control‐Dead	−1.94	0.26	−7.38	**< 0.001**
Control‐Live	−2.85	0.27	−10.75	**< 0.001**
Dead‐Live	−0.91	0.21	−4.24	**< 0.001**
*Post‐recruit richness*				
Control‐Dead	−1.81	0.2	−8.86	**< 0.001**
Control‐Live	−2.45	0.2	−12.21	**< 0.001**
Dead‐Live	−0.64	0.13	−4.94	**< 0.001**

**TABLE A7:** Results of PERMANOVA for the Hellinger‐transformed abundance data of recruit and post‐recruit assemblages. Bold *p*‐values indicate significant effects (*p* < 0.05). Permutations were stratified within months and constrained by block (each block contained one live, one dead, and one control patch). Patch represents the three habitat conditions (live, dead, control).

Stage	Factor	Df	Sum of Sqs	*R* ^2^	*F*	Pr (> *F*)
Recruits	Patch	2	1.22	0.05	1.48	**0.006**
	Block	1	0.43	0.02	1.05	0.294
	Residual	53	21.8	0.93		
	Total	56	23.45	1		
Post‐recruit	Patch	2	5.38	0.16	7.86	**0.001**
	Block	1	0.68	0.02	1.98	**0.010**
	Residual	79	27.05	0.82		
	Total	82	33.11	1		

**TABLE A8:** Results of pairwise PERMANOVA for the Hellinger transformed abundance count data matrix of recruit and post‐recruit assemblages comparing pairs of conditions in the live coral, dead coral, and control patches. Bold letters in the *p*‐value column indicate statistical significance (*p* < 0.05). We stratified permutations within months.

Stage	Pair		Df	Sum of Sqs	*R* ^2^	*F*	Pr (> F)
Recruits	Live × Dead		1	0.77	0.04	1.88	**0.033**
		Residual	51	20.87	0.96		
		Total	52	21.64	1		
	Live × Control		1	0.52	0.04	1.29	0.178
		Residual	29	11.61	0.96		
		Total	30	12.12	1		
	Dead × Control		1	0.42	0.03	0.97	0.506
		Residual	28	12	0.97		
		Total	29	12.42	1		
Post‐recruits	Live × Dead		1	3.65	0.14	11.04	**0.001**
		Residual	67	22.13	0.86		
		Total	68	25.78	1		
	Live × Control		1	2.89	0.17	9.52	**0.001**
		Residual	48	14.57	0.83		
		Total	49	17.46	1		
	Dead × Control		1	1.08	0.05	2.59	**0.001**
		Residual	45	18.75	0.95		
		Total	46	19.82	1		

**TABLE A9:** GLMM outputs for the comparison of recruit and post‐recruit abundances by mobility habit. The estimated coefficients (log scale), standard errors, 95% confidence intervals, *z*‐values, and *p*‐values of fixed effects are presented. Significant differences (*p* < 0.05) are highlighted in bold.

	Estimate	SE	Lower limit	Upper limit	*z*	Pr (>|z|)
*Recruit/high mobility*						
Control (Intercept)	−1.59	0.51	−2.59	−0.59	−3.12	**< 0.01**
Dead	2.11	0.44	1.25	2.98	4.79	**< 0.001**
Live	2.13	0.44	1.27	2.99	4.85	**< 0.001**
*Recruit/low mobility*						
Control (Intercept)	−1.79	0.83	−3.42	−0.17	−2.16	**0.031**
Dead	1	0.9	−0.77	2.77	1.11	0.267
Live	1.04	0.9	−0.72	2.81	1.16	0.247
*Post‐recruit/high mobility*						
Control (Intercept)	−0.41	0.27	−0.95	0.12	−1.52	0.129
Dead	1.93	0.3	1.35	2.51	6.53	**< 0.001**
Live	2.42	0.29	1.85	3	8.25	**< 0.001**
*Post‐recruit/low mobility*						
Control (Intercept)	0.09	0.34	−0.58	0.76	0.27	0.791
Dead	−0.6	0.42	−1.42	0.21	−1.45	0.148
Live	0.58	0.41	−0.22	1.38	1.43	0.154

**TABLE A10:** Post hoc pairwise comparisons of recruit and post‐recruit abundances by mobility habit. Estimated marginal means contrasts are presented with log‐scale estimates, standard error, *z*‐ratio, and Tukey‐adjusted *p*‐values. Significant differences (*p* < 0.05) are highlighted in bold. Tests are based on Wald *z*‐statistics with asymptotic standard errors (df = ∞).

	Estimate	SE	*z*.ratio	*p*
*Recruits/high mobility*				
Control—Dead	−2.11	0.44	−4.79	**< 0.001**
Control—Live	−2.13	0.44	−4.85	**< 0.001**
Dead—Live	−0.02	0.32	−0.06	1
*Recruits/low mobility*				
Control—Dead	−3.11	0.8	−3.91	**< 0.001**
Control—Live	−3.17	0.8	−3.98	**< 0.001**
Dead—Live	−0.06	0.37	−0.16	0.99
*Post‐recruits/high mobility*				
Control—Dead	−1.93	0.3	−6.53	**< 0.001**
Control—Live	−2.42	0.29	−8.25	**< 0.001**
Dead—Live	−0.49	0.23	−2.16	0.08
*Post‐recruits/low mobility*				
Control—Dead	−1.33	0.3	−4.41	**< 0.001**
Control—Live	−3	0.29	−10.25	**< 0.001**
Dead—Live	−1.67	0.24	−7.11	**< 0.001**

**TABLE A11:** GLMM outputs for the comparison of recruit abundance by trophic group. The estimated coefficients (log scale), standard errors, 95% confidence intervals, *z*‐values, and *p*‐values of fixed effects are presented. Significant differences (*p* < 0.05) are highlighted in bold. Standard errors and confidence intervals exceeding ±100 were truncated for readability.

Recruits	Trophic group	Estimate	SE	Lower limit	Upper limit	*z*	Pr (>|z|)
Control (Intercept)	CAR	−4.01	1.1	−6.17	−1.85	−3.64	**< 0.001**
Dead	CAR	0.64	1.29	−1.88	3.16	0.5	0.62
Live	CAR	2.82	1.09	0.67	4.96	2.58	**0.01**
Control (Intercept)	CLN	−0.08	1.47	−2.95	2.8	−0.05	0.96
Dead	CLN	0.14	1.82	−3.42	3.7	0.08	0.94
Live	CLN	−1.51	1.61	−4.67	1.64	−0.94	0.35
Control (Intercept)	MIF	1.26	1.18	−1.06	3.58	1.06	0.29
Dead	MIF	2.27	1.43	−0.54	5.07	1.58	0.11
Live	MIF	0.28	1.26	−2.19	2.75	0.22	0.83
Control (Intercept)	OMN	−16.84	> 100	< −100	> 100	0	1
Dead	OMN	17.35	> 100	< −100	> 100	0	1
Live	MON	15.66	> 100	< −100	> 100	0	1
Control (Intercept)	PLK	0.6	1.28	−1.91	3.12	0.47	0.64
Dead	PLK	2.02	1.52	−0.97	5	1.32	0.19
Live	PLK	−0.1	1.37	−2.78	2.58	−0.07	0.94
Control (Intercept)	RH	1.8	1.14	−0.43	4.03	1.58	0.11
Dead	RH	1.09	1.4	−1.64	3.83	0.78	0.43
Live	RH	−2.61	1.28	−5.11	−0.11	−2.05	**0.04**

Abbreviations: CAR, carnivore; CLN, cleaner; COR, corallivore; MIF, motile invertebrate feeder; OMN, omnivore; PLK, planktivore; RH, roving herbivore; SIF, sessile invertebrate feeder; TH, territorial herbivore.

**TABLE A12:** Post hoc pairwise comparisons of recruit abundance by trophic group. Estimated marginal means contrasts are presented with log‐scale estimates, standard error, *z*‐ratio, and Tukey‐adjusted *p*‐values. Significant differences (*p* < 0.05) are highlighted in bold. Tests are based on Wald *z*‐statistics with asymptotic standard errors (df = ∞). Standard errors exceeding ±100 were truncated for readability.

Recruits	Trophic group	Estimate	SE	*z*.ratio	*p*
Control—Dead	CAR	−0.64	1.29	−0.5	0.87
Control—Live	CAR	−2.82	1.09	−2.58	**0.03**
Dead—Live	CAR	−2.18	0.83	−2.62	**0.02**
Control—Dead	CLN	−0.77	1.28	−0.6	0.82
Control—Live	CLN	−1.3	1.18	−1.1	0.51
Dead—Live	CLN	−0.53	0.95	−0.56	0.84
Control—Dead	MIF	−2.9	0.63	−4.59	**< 0.001**
Control—Live	MIF	−3.09	0.63	−4.92	**< 0.001**
Dead—Live	MIF	−0.19	0.37	−0.51	0.87
Control—Dead	OMN	−17.99	< 100	0	1
Control—Live	OMN	−18.48	< 100	0	1
Dead—Live	OMN	−0.49	0.8	−0.61	0.82
Control—Dead	PLK	−2.65	0.82	−3.25	**< 0.001**
Control—Live	PLK	−2.72	0.82	−3.31	**< 0.001**
Dead—Live	PLK	−0.06	0.43	−0.15	0.99
Control—Dead	RH	−1.73	0.55	−3.16	**< 0.001**
Control—Live	RH	−0.21	0.65	−0.32	0.95
Dead—Live	RH	1.52	0.54	2.81	**0.01**

Abbreviations: CAR, carnivore; CLN, cleaner; COR, corallivore; MIF, motile invertebrate feeder; OMN, omnivore; PLK, planktivore; RH, roving herbivore; SIF, sessile invertebrate feeder; TH, territorial herbivore.

**TABLE A13:** GLMM outputs for the comparison of post‐recruit abundance by trophic group. The estimated coefficients (log scale), standard errors, 95% confidence intervals, *z*‐values, and *p*‐values of fixed effects are presented. Significant differences (*p* < 0.05) are highlighted in bold. Standard errors and confidence intervals exceeding ±100 were truncated for readability.

Post‐recruit	Trophic group	Estimate	SE	Lower limit	Upper limit	*z*	Pr (>|z|)
Control (Intercept)	CAR	−2.54	0.61	−3.74	−1.34	−4.16	**< 0.001**
Dead	CAR	1.91	0.66	0.62	3.21	2.89	**< 0.001**
Live	CAR	4.01	0.63	2.78	5.25	6.38	**< 0.001**
Control (Intercept)	CLN	−1.11	1.18	−3.42	1.2	−0.94	0.35
Dead	CLN	−0.84	1.35	−3.49	1.81	−0.62	0.54
Live	CLN	−1.21	1.23	−3.62	1.2	−0.98	0.33
Control (Intercept)	COR	−17.39	> 100	< −100	> 100	−0.01	1
Dead	COR	−14.87	> 100	< −100	> 100	0	1
Live	COR	12.3	> 100	< −100	> 100	0	1
Control (Intercept)	MIF	1.94	0.66	0.65	3.24	2.95	**< 0.001**
Dead	MIF	−0.02	0.74	−1.47	1.42	−0.03	0.98
Live	MIF	−1.69	0.71	−3.07	−0.31	−2.4	**< 0.001**
Control (Intercept)	OMN	2.05	0.66	0.76	3.34	3.12	**< 0.001**
Dead	OMN	−1.28	0.75	−2.75	0.19	−1.71	0.09
Live	OMN	−2.4	0.71	−3.79	−1.01	−3.38	**< 0.001**
Control (Intercept)	PLK	0.47	0.77	−1.04	1.97	0.61	0.54
Dead	PLK	0.19	0.85	−1.48	1.85	0.22	0.83
Live	PLK	−0.04	0.81	−1.62	1.55	−0.05	0.96
Control (Intercept)	RH	−0.43	0.94	−2.28	1.42	−0.45	0.65
Dead	RH	0.66	1.02	−1.33	2.66	0.65	0.51
Live	RH	−1.28	0.99	−3.22	0.67	−1.29	0.2
Control (Intercept)	SIF	−19.2	> 100	< −100	> 100	0	1
Dead	SIF	16.88	> 100	< −100	> 100	0	1
Live	SIF	16.85	> 100	< −100	> 100	0	1
Control (Intercept)	TH	−18.41	> 100	< −100	> 100	0	1
Dead	TH	16.81	> 100	< −100	> 100	0	1
Live	TH	14.67	> 100	< −100	> 100	0	1

Abbreviations: CAR, carnivore; CLN, cleaner; COR, corallivore; MIF, motile invertebrate feeder; OMN, omnivore; PLK, planktivore; RH, roving herbivore; SIF, sessile invertebrate feeder; TH, territorial herbivore.

**TABLE A14:** Post hoc pairwise comparisons of post‐recruit abundance by trophic group. Estimated marginal means contrasts are presented with log‐scale estimates, standard error, *z*‐ratio, and Tukey‐adjusted *p*‐values. Significant differences (*p* < 0.05) are highlighted in bold. Tests are based on Wald *z*‐statistics with asymptotic standard errors (df = ∞). Standard errors exceeding ±100 were truncated for readability.

Post‐recruit	Trophic group	Estimate	SE	*z*.ratio	*p*
Control—Dead	CAR	−1.91	0.66	−2.89	**0.01**
Control—Live	CAR	−4.01	0.63	−6.38	**< 0.001**
Dead—Live	CAR	−2.1	0.33	−6.29	**< 0.001**
Control—Dead	CLN	−1.08	1.18	−0.91	0.63
Control—Live	CLN	−2.8	1.06	−2.65	**0.02**
Dead—Live	CLN	−1.73	0.67	−2.58	**0.03**
Control—Dead	COR	12.95	< 100	0	1
Control—Live	COR	−16.31	< 100	0	1
Dead—Live	COR	−29.26	< 100	0	1
Control—Dead	MIF	−1.89	0.33	−5.81	**< 0.001**
Control—Live	MIF	−2.32	0.32	−7.18	**< 0.001**
Dead—Live	MIF	−0.43	0.25	−1.69	0.21
Control—Dead	OMN	−0.63	0.35	−1.79	0.17
Control—Live	OMN	−1.62	0.33	−4.89	**< 0.001**
Dead—Live	OMN	−0.99	0.3	−3.33	**< 0.001**
Control—Dead	PLK	−2.1	0.53	−3.97	**< 0.001**
Control—Live	PLK	−3.97	0.51	−7.77	**< 0.001**
Dead—Live	PLK	−1.87	0.29	−6.48	**< 0.001**
Control—Dead	RH	−2.58	0.77	−3.34	**< 0.001**
Control—Live	RH	−2.74	0.77	−3.57	**< 0.001**
Dead—Live	RH	−0.16	0.35	−0.45	0.9
Control—Dead	SIF	−18.8	< 100	0	1
Control—Live	SIF	−20.86	< 100	0	1
Dead—Live	SIF	−2.07	0.79	−2.62	**0.02**
Control—Dead	TH	−18.72	< 100	0	1
Control—Live	TH	−18.68	< 100	0	1
Dead—Live	TH	0.04	0.75	0.06	1

Abbreviations: CAR, carnivore; CLN, cleaner; COR, corallivore; MIF, motile invertebrate feeder; OMN, omnivore; PLK, planktivore; RH, roving herbivore; SIF, sessile invertebrate feeder; TH, territorial herbivore.
